# The C-Reactive Protein/Albumin Ratio Predicts In-Hospital Mortality Across Age Groups

**DOI:** 10.3390/nu18182988

**Published:** 2026-09-12

**Authors:** Cristiano Capurso, Aurelio Lo Buglio, Francesco Bellanti, Giuseppe Di Gioia, Gaetano Serviddio

**Affiliations:** 1 Department of Medical and Surgical Sciences, University of Foggia, Viale Luigi Pinto 1, 71122 Foggia, Italy; aurelio.lobuglio@unifg.it (A.L.B.); francesco.bellanti@unifg.it (F.B.); gaetano.serviddio@unifg.it (G.S.); 2 Medical Clinic Unit, Policlinico Riuniti, Viale Luigi Pinto 1, 71122 Foggia, Italy; giuseppe.digioia@unifg.it

**Keywords:** C-reactive protein/albumin ratio, chronological age, in-hospital mortality, inflammation, risk stratification, prognostic biomarker

## Abstract

**Background:** The C-reactive protein-to-albumin (CRP/Alb) ratio integrates systemic inflammation and nutritional status and has emerged as a promising prognostic biomarker in hospitalized patients. However, because chronological age is itself a major determinant of mortality, it remains unclear whether the prognostic performance of the CRP/Alb ratio is influenced by age. **Objectives:** This study evaluated the prognostic performance of the CRP/Alb ratio for 30-day and 7-day in-hospital mortality, assessing the incremental contribution of chronological age and whether the association and discriminative performance of the CRP/Alb ratio differed across age groups. **Methods:** We retrospectively analyzed 3791 consecutive adult patients admitted to the Acute Care Unit of Clinical Medicine (formerly Internal and Aging Medicine) of the Policlinico Riuniti University Hospital, Foggia, Italy, between 2019 and 2025. The primary outcome was 30-day in-hospital mortality, whereas 7-day in-hospital mortality was considered a secondary outcome. Prognostic performance was evaluated using receiver operating characteristic (ROC) curve analysis, multivariable logistic regression, age-adjusted models, age-stratified ROC comparisons, and interaction analyses. **Results:** Among 3791 patients, 542 (14.3%) died within 30 days of hospitalization and 240 (6.3%) within 7 days. For 30-day mortality, the CRP/Alb ratio showed an AUC of 0.733 (95% CI 0.712–0.754), which increased to 0.759 (95% CI 0.739–0.779) after incorporating chronological age into the model (*p* < 0.001). For 7-day mortality, the corresponding AUCs were 0.752 (95% CI 0.724–0.781) and 0.768 (95% CI 0.740–0.796), respectively (*p* = 0.005). In multivariable logistic regression, each doubling of the CRP/Alb ratio was associated with higher odds of both 30-day mortality (OR = 1.484, 95% CI 1.409–1.563) and 7-day mortality (OR = 1.578, 95% CI 1.457–1.710), independently of chronological age. No significant interaction between ln(CRP/Alb ratio) and age was observed for either 30-day (*p* = 0.322) or 7-day mortality (*p* = 0.491), and age-stratified ROC curves did not differ significantly across age groups. **Conclusions:** The CRP/Alb ratio was independently associated with both 30-day and 7-day in-hospital mortality, with discriminative performance largely preserved across age groups. Chronological age provided a modest but statistically significant improvement in discrimination but did not significantly modify the association between the CRP/Alb ratio and mortality. These findings support the CRP/Alb ratio as a readily available marker for short-term risk stratification in hospitalized adults, while external validation is required before its use as a stand-alone clinical prediction tool.

## 1. Introduction

Population ageing has substantially changed the clinical profile of patients requiring hospital care worldwide [1]. With advancing age, chronic diseases, frailty, functional limitations, and disability become increasingly prevalent [2,3,4], contributing to greater healthcare utilization and recurrent hospitalization [5]. Older adults admitted to acute medical units are therefore a particularly heterogeneous population, in whom multimorbidity, acute disease, functional impairment, polypharmacy, and nutritional and inflammatory disturbances frequently coexist [6,7,8,9,10,11,12,13]. These characteristics contribute to the substantial short- and long-term mortality observed after acute hospitalization [14,15] and emphasize the need for simple tools capable of identifying patients at increased risk early in the course of admission.

Inflammation and nutritional impairment are closely interconnected during acute and chronic illness. A pronounced inflammatory response promotes metabolic and catabolic changes and is frequently accompanied by declining serum albumin concentrations, functional deterioration, and poorer clinical outcomes. At the same time, malnutrition may impair immune competence and has been associated with longer hospital stays, recurrent hospitalization, and reduced survival [16,17,18,19,20]. Disease-related malnutrition is particularly relevant in hospitalized and older populations, in whom inadequate intake, loss of appetite, diagnostic fasting, medication-related effects, gastrointestinal dysfunction, and other hospitalization-related factors may further compromise nutritional status [21]. When sustained, this combination of inflammation and catabolism may contribute to muscle loss, impaired recovery, treatment failure, and increased mortality [22].

These processes may be especially important in older patients. Acute illness frequently occurs on a background of frailty, multimorbidity, altered immune responses, and reduced physiological reserve [23,24]. Ageing is also associated with diminished metabolic resilience, limiting the capacity to adapt to nutritional, energetic, and inflammatory challenges [25,26]. Although nutritional intervention may help restore metabolic balance [27], severe inflammation can attenuate the response to nutritional support through alterations in protein, lipid, and carbohydrate metabolism [28,29,30,31,32,33]. Inflammatory pathways may also contribute directly to disease-related malnutrition and functional decline [34,35,36,37], and nutritional deterioration in hospitalized patients frequently accompanies changes in inflammatory or infection-related biomarkers [38].

Among routinely available laboratory markers, C-reactive protein (CRP) provides an established measure of the systemic inflammatory response and has been associated with mortality and other adverse outcomes in several acute and chronic conditions [39,40,41,42,43]. Serum albumin, in contrast, is influenced by nutritional status, inflammation, and disease severity; low concentrations have repeatedly been associated with mortality, prolonged hospitalization, and complications in older inpatients [44,45,46,47,48,49,50,51]. The concurrent occurrence of elevated CRP and reduced albumin has also been linked to poorer outcomes in patients with marked systemic inflammation [52,53,54].

Combining these two measurements into the C-reactive protein-to-albumin ratio (CRP/Alb ratio) provides a single index incorporating information related to both inflammatory activity and nutritional/metabolic status. Previous investigations have associated higher CRP/Alb ratios with adverse outcomes in sepsis and other infectious conditions [55,56,57,58], as well as in non-septic clinical populations [59,60,61,62]. Associations have also been reported in acute and chronic heart failure, where the ratio has been related to mortality, rehospitalization, disease severity, and clinical features such as frailty and cachexia [63,64,65,66,67]. More broadly, studies in critically ill and hospitalized populations support its potential role as a readily obtainable prognostic marker [68,69,70,71]. In our previous work, the CRP/Alb ratio was associated with early in-hospital mortality among older medical inpatients, with particularly strong discrimination for deaths occurring during the first week of hospitalization.

An unresolved issue is whether the prognostic information provided by the CRP/Alb ratio is influenced by chronological age. Age is itself strongly associated with mortality among hospitalized adults and is accompanied by changes in immune function, inflammatory regulation, nutritional status, and physiological reserve. Consequently, age could either provide prognostic information additional to that conveyed by the CRP/Alb ratio or modify the relationship between the biomarker and mortality. Despite the growing literature on the CRP/Alb ratio, this question has received comparatively limited investigation.

Accordingly, this retrospective, observational, single-center study evaluated the prognostic performance of the CRP/Alb ratio for 30-day and 7-day in-hospital mortality across the adult age spectrum in a large cohort of hospitalized adults admitted to the Acute Care Unit of Clinical Medicine (formerly Internal and Aging Medicine) of the Policlinico Riuniti University Hospital in Foggia, Italy. We specifically examined whether chronological age added discriminative information to the CRP/Alb ratio and whether either its association with mortality or its discriminative performance differed across predefined age groups.

## 2. Materials and Methods

### 2.1. Study Population

We conducted and reported our study in line with the TRIPOD recommendations [72]. A complete TRIPOD checklist is available in the Supplementary Materials.

We retrospectively examined 4895 consecutive patients admitted to the Acute Care Unit of Clinical Medicine (formerly Internal and Aging Medicine), Policlinico Riuniti di Foggia, between 1 January 2019 and 31 December 2025. Eligible patients were adults aged ≥18 years with available CRP and serum albumin measurements at admission and ascertainable 30-day hospital outcomes. Patients were excluded if they were aged <18 years, were receiving albumin infusions at admission, left the hospital against medical advice, were transferred to another department or acute care facility, were discharged to a long-term care institution, or had missing admission laboratory data required for the analysis.

**Figure 1:**
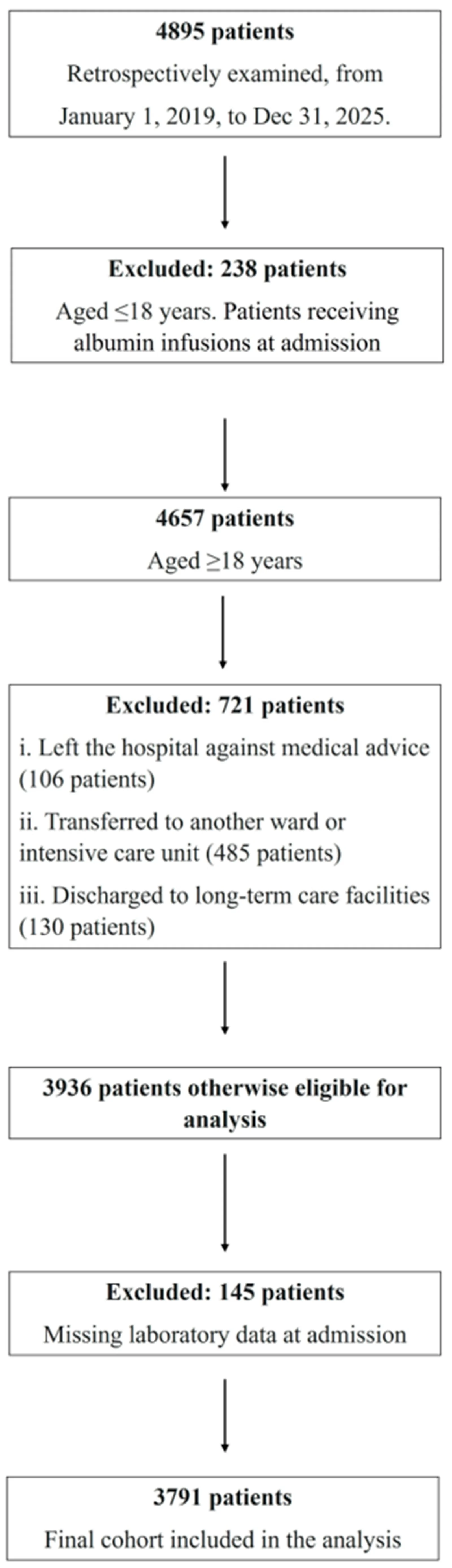
Flowchart steps from the starting population to the final cohort included in the analysis.

Of the 4895 patients initially assessed, 238 were excluded because they were aged < 18 years or were receiving albumin infusions at admission. A further 106 patients left the hospital against medical advice, 485 were transferred to another ward or intensive care unit, and 130 were discharged to long-term care facilities. Finally, 145 patients with missing admission laboratory data were excluded. The final analytical cohort comprised 3791 patients. The flowchart steps are summarized in Figure 1.

For all patients included in the study, the following parameters were considered: serum C-reactive protein (CRP) and albumin values measured at admission, length of stay (LOS), and hospital outcome (discharge or death). The primary outcome of the study was 30-day in-hospital mortality. A secondary outcome was 7-day in-hospital mortality, which was evaluated to investigate the ability of the CRP/Alb ratio to identify patients at particularly high risk of very early death after admission.

### 2.2. Statistical Analysis

The Kolmogorov–Smirnov test was used to assess the distribution of continuous variables. Because the examined continuous variables did not follow a normal distribution (*p* < 0.001), they are reported as median and interquartile range [IQR], and between-group comparisons were performed using the nonparametric Mann–Whitney U test. Monte Carlo significance estimates for the Mann–Whitney U test were based on 3791 samples, with a 99% confidence level. Spearman’s rank correlation coefficient was used to assess correlations between continuous variables, without Monte Carlo estimation. The chi-square test was used to compare categorical variables between groups (males vs. females and deceased vs. non-deceased), with Monte Carlo significance estimates based on 3791 samples and a 99% confidence level. For continuous variables compared using the Mann–Whitney U test, effect size was quantified as r = |z|/√N, where z is the standardized test statistic, and N is the total number of observations included in the comparison. Absolute r values of approximately 0.10, 0.30, and 0.50 were interpreted as small, moderate, and large effects, respectively. Cohen’s h was used to quantify effect size for differences between two proportions, whereas the Phi coefficient was used for 2 × 2 categorical comparisons [73,74].

The predictive performance of the CRP/Alb ratio for 30-day in-hospital mortality, the study’s primary outcome, was initially assessed using receiver operating characteristic (ROC) curve analysis, with calculation of the area under the curve (AUC) and corresponding 95% confidence intervals. The optimal cut-off value was identified using the Youden index. Thirty-day mortality was defined as death occurring during hospitalization within 30 days of admission; patients who remained alive beyond 30 days were classified as non-events for this outcome.

Because the CRP/Alb ratio showed a markedly right-skewed distribution, its natural logarithm [ln(CRP/Alb ratio)] was used when the ratio was entered as a continuous predictor in multivariable logistic regression models. To assess the incremental discriminative contribution of chronological age, predicted probabilities were derived from logistic regression models including ln(CRP/Alb ratio) and age at admission. ROC curves derived from these predicted probabilities were compared with those of the CRP/Alb ratio alone using statistical comparison of the corresponding AUCs. For clinical interpretability, the association of the CRP/Alb ratio with mortality was expressed as the odds ratio (OR) associated with a doubling of the ratio, whereas the effect of age was expressed per 10-year increase.

To assess whether chronological age modified the association between the CRP/Alb ratio and mortality, interaction terms between ln(CRP/Alb ratio) and age were tested in multivariable logistic regression models. In addition, the discriminative performance of the CRP/Alb ratio was evaluated across predefined age groups (<65, 65–74, 75–84, and ≥85 years), and age-stratified ROC curves were statistically compared.

Secondary analyses were performed considering 7-day in-hospital mortality as an additional outcome. Seven-day mortality was defined as death occurring during hospitalization within 7 days of admission; patients who remained alive beyond 7 days were classified as non-events for this outcome. ROC analysis, determination of the optimal cut-off value, age-adjusted logistic regression and ROC analysis, AUC comparisons, age-stratified ROC analysis, and interaction analysis were repeated using 7-day mortality as the dependent variable.

Model calibration was assessed using the Hosmer–Lemeshow goodness-of-fit test, comparison of observed and expected events across deciles of predicted risk, the Brier score, and graphical assessment of observed versus mean predicted mortality across deciles of predicted risk.

The statistical packages IBM SPSS version 25 (Armonk, NY, USA) and STATA SE 14.2 (College Station, TX, USA) were used. *p*-values lower than 0.05 were considered statistically significant.

### 2.3. Missing-Data Analysis and Sensitivity Analysis

The primary analyses were based on complete cases with available admission CRP and serum albumin measurements. To assess the potential impact of this exclusion, patients with and without available CRP/Alb ratio data were compared with respect to age, sex, LOS, and 30-day in-hospital mortality. In addition, we performed a multiple-imputation sensitivity analysis in the 3936 patients otherwise eligible for the 30-day analysis, including the 145 patients excluded from the primary analysis because CRP/Alb ratio data were unavailable. Missing CRP and serum albumin values were imputed using multiple imputation by chained equations with predictive mean matching (k = 5), using 50 imputations. Sex, which was missing in one patient, was imputed using logistic regression. The imputation model included age, sex, overall, in-hospital mortality, and LOS. Two serum albumin values recorded as zero, which rendered calculation of the ratio unusable, were treated as missing for the imputation sensitivity analysis. The CRP/Alb ratio and its natural logarithm were derived after imputation. Logistic-regression estimates for 30-day in-hospital mortality were combined according to Rubin’s rules. As a descriptive assessment of discrimination, the AUC was calculated separately in each imputed dataset, and the mean AUC across the 50 imputed datasets was reported; AUC estimates were not combined using Rubin’s rules.

### 2.4. Internal Validation

Internal validation of the age-adjusted 7-day and 30-day mortality models was performed using nonparametric bootstrap resampling with 1000 repetitions. In each bootstrap sample, the complete model was refitted, and its discriminative performance was evaluated in both the bootstrap sample and the original dataset. Optimism was estimated as the mean difference between the AUC obtained in each bootstrap sample and the AUC obtained when the corresponding bootstrap-fitted model was applied to the original dataset. The mean optimism was subtracted from the apparent AUC to obtain the optimism-corrected AUC. Optimism-corrected calibration slopes and intercepts were derived using the same bootstrap resampling framework.

## 3. Results

### 3.1. General Characteristics of the Study Cohort

Female participants were significantly older than male participants (median [IQR], 81 [72–87] vs. 76 [65–84] years; *p* < 0.001). No statistically significant differences between sexes were observed in length of stay (9 [6–14] vs. 9 [6–14] days; *p* = 0.622), serum albumin (3.06 [2.58–3.47] vs. 3.09 [2.58–3.52] g/dL; *p* = 0.079), CRP (36.6 [8.7–118.9] vs. 43.1 [10.4–126.5] mg/L; *p* = 0.065), or CRP/albumin ratio (12.37 [2.53–42.88] vs. 14.50 [3.04–45.72]; *p* = 0.130). Detailed sex-stratified characteristics are summarized in Table 1.

### 3.2. Age Stratification

To further investigate age-related effects, patients were stratified, according to commonly adopted geriatric classifications [75,76], into age categories as follows: “young” (<65 years), “young-old” (65–74 years), “old” (75–84 years), and “very old” (≥85 years). Men were more prevalent in the “young” and “young-old” groups (*p* < 0.001), while women were predominant in the “very old” category (*p* < 0.001). No significant sex difference was observed in the “old” group (*p* = 0.626). Table 2 summarizes detailed age-stratified characteristics.

### 3.3. Mortality and Associated Variables

Patients who died were significantly older than those who survived (median [IQR], 84 [76–88] vs. 77 [67–85] years; *p* < 0.001). There was no significant difference in mortality according to sex (*p* = 0.552), and length of stay did not differ significantly between deceased and non-deceased patients (9 [4–16] vs. 9 [6–14] days; *p* = 0.163). Deceased patients had significantly lower serum albumin concentrations (2.50 [2.13–2.93] vs. 3.15 [2.71–3.56] g/dL; *p* < 0.001) and significantly higher CRP concentrations (106.5 [43.2–180.6] vs. 31.9 [7.2–105.4] mg/L; *p* < 0.001) and CRP/albumin ratios (42.74 [15.24–81.53] vs. 10.18 [2.11–36.54]; *p* < 0.001). Descriptive statistics by survival status are shown in Table 3.

### 3.4. Discriminative Performance of the CRP/Alb Ratio and Incremental Contribution of Age

**Figure 2:**
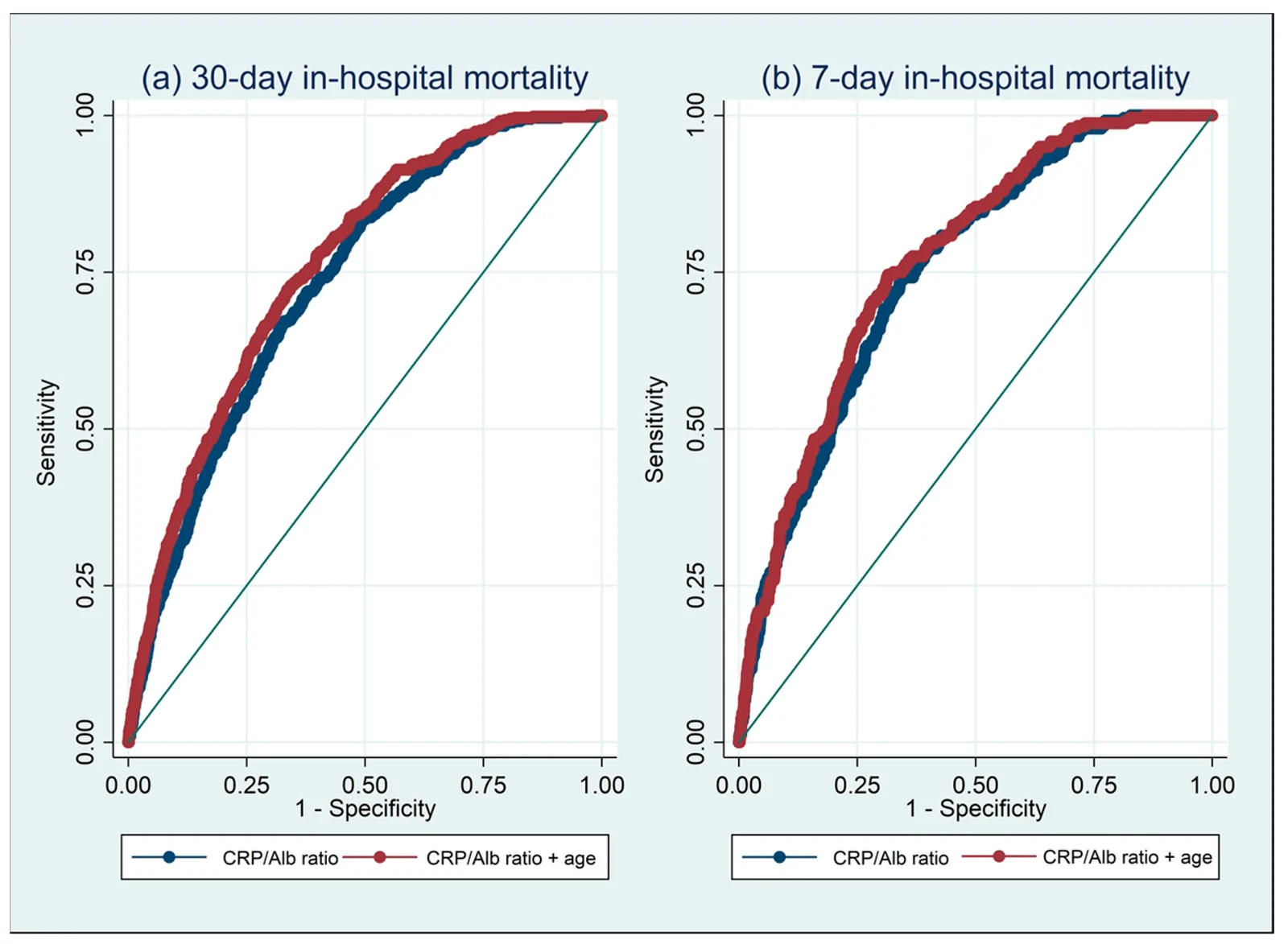
ROC curves for the prediction of in-hospital mortality. (**a**) ROC curves for 30-day in-hospital mortality comparing the CRP/Alb ratio alone with the model including ln(CRP/Alb ratio) and chronological age. The AUC increased from 0.733 to 0.759 (*p* < 0.001). (**b**) ROC curves for 7-day in-hospital mortality comparing the same models. The AUC increased from 0.752 to 0.768 (*p* = 0.005).

As shown in Figure 2a, for 30-day in-hospital mortality, the CRP/Alb ratio showed moderate discriminative ability, with an AUC of 0.733 (95% CI 0.712–0.754). The optimal cut-off identified by the Youden index was 25.00, corresponding to a sensitivity of 66.6%, a specificity of 67.7%, and a Youden index of 0.343. After incorporating chronological age into the model together with ln(CRP/Alb ratio), discrimination significantly improved to an AUC of 0.759 (95% CI 0.739–0.779), representing an absolute increase in AUC of 0.026 compared with the CRP/Alb ratio alone (χ^2^ = 22.44, *p* < 0.001).

As shown in Figure 2b, for 7-day in-hospital mortality, the CRP/Alb ratio yielded an AUC of 0.752 (95% CI 0.724–0.781). The optimal cut-off identified by the Youden index was 25.92, corresponding to a sensitivity of 73.8%, a specificity of 65.9%, and a Youden index of 0.396. The model including ln(CRP/Alb ratio) and chronological age achieved an AUC of 0.768 (95% CI 0.740–0.796), corresponding to an absolute increase in AUC of 0.016 compared with the CRP/Alb ratio alone (χ^2^ = 7.76, *p* = 0.005). The discrimination results for both mortality outcomes are summarized in Table 4.

#### 3.4.1. 30-Day In-Hospital Mortality Risk

As shown in Table 5, age-stratified ROC analyses indicated that the discriminative performance of the CRP/Alb ratio was broadly maintained across age groups. The AUC was 0.763 (95% CI 0.704–0.823) in patients aged <65 years, 0.749 (95% CI 0.694–0.804) in those aged 65–74 years, 0.732 (95% CI 0.695–0.769) in those aged 75–84 years, and 0.699 (95% CI 0.663–0.734) in patients aged ≥85 years. Although a numerical decrease in discrimination was observed with increasing age, the overall comparison of AUCs across age groups was not statistically significant (χ^2^(3) = 4.51, *p* = 0.211). Thus, the observed numerical pattern did not provide evidence of a statistically significant age-related difference in the discriminative performance of the CRP/Alb ratio.

In the multivariable logistic regression model, the natural logarithm of the CRP/Alb ratio was significantly associated with 30-day in-hospital mortality after adjustment for chronological age (β = 0.569, *p* < 0.001). Expressed on a clinically interpretable scale, each doubling of the CRP/Alb ratio was associated with a 48.4% increase in the odds of 30-day mortality (OR = 1.484, 95% CI 1.409–1.563). Age was also independently associated with mortality (OR per 10-year increase = 1.459, 95% CI 1.336–1.593; *p* < 0.001).

As summarized in Table 6, no statistically significant interaction between ln(CRP/Alb ratio) and chronological age was observed (β = −0.0342, 95% CI −0.1018 to 0.0335; *p* = 0.322), providing no evidence that the association between the CRP/Alb ratio and 30-day mortality varied significantly according to chronological age.

#### 3.4.2. 7-Day In-Hospital Mortality Risk

Concerning 7-day in-hospital mortality, the AUC of the CRP/Alb ratio was 0.776 (95% CI 0.690–0.862) in patients aged <65 years, 0.765 (95% CI 0.686–0.843) in those aged 65–74 years, 0.766 (95% CI 0.717–0.816) in those aged 75–84 years, and 0.710 (95% CI 0.663–0.758) in patients aged ≥85 years, as summarized in Table 7. The overall comparison showed no statistically significant differences in discriminative performance across age groups (χ^2^(3) = 3.52, *p* = 0.318).

In the multivariable logistic regression model, the natural logarithm of the CRP/Alb ratio was significantly associated with 7-day in-hospital mortality after adjustment for chronological age (β = 0.658, *p* < 0.001). Each doubling of the CRP/Alb ratio was associated with a 57.8% increase in the odds of 7-day mortality (OR = 1.578, 95% CI 1.457–1.710). Age was also independently associated with mortality (OR per 10-year increase = 1.331, 95% CI 1.180–1.502; *p* < 0.001).

As summarized in Table 8, no statistically significant interaction between ln(CRP/Alb ratio) and chronological age was observed (β = −0.0357, 95% CI −0.1373 to 0.0659; *p* = 0.491), providing no evidence that the association between the CRP/Alb ratio and 7-day mortality varied significantly according to chronological age.

Model calibration was satisfactory for both outcomes. For 30-day in-hospital mortality, the Hosmer–Lemeshow goodness-of-fit test showed no significant evidence of lack of fit (χ^2^(8) = 10.91, *p* = 0.207), with a Brier score of 0.1095. Similarly, for 7-day in-hospital mortality, the Hosmer–Lemeshow test was non-significant (χ^2^(8) = 8.74, *p* = 0.365), with a Brier score of 0.0558. Calibration plots based on deciles of predicted risk showed generally close agreement between observed and predicted mortality across the risk distribution (Figures S2 and S3; Tables S1 and S2).

#### 3.4.3. Sensitivity Analysis for Missing Data

Among the 3936 patients otherwise eligible for the 30-day analysis, 145 (3.7%) had unavailable CRP/albumin ratio data and were excluded from the primary complete-case analysis. Compared with complete cases, patients with missing CRP/albumin ratio data had substantially higher 30-day in-hospital mortality (54.5% vs. 14.3%, *p* < 0.001) and a shorter length of stay (median 2 [IQR 1–7] vs. 9 [6–14] days, *p* < 0.001). In contrast, age (median 78 [68–86] years in both groups, *p* = 0.779) and sex distribution (female, 50.7% vs. 49.5%, *p* = 0.785) were similar between groups (Table S4).

In the multiple-imputation sensitivity analysis including all 3936 patients, the association between the CRP/albumin ratio and 30-day in-hospital mortality remained materially unchanged. Each doubling of the CRP/albumin ratio was associated with a 46.6% increase in the odds of 30-day mortality (OR = 1.466, 95% CI 1.391–1.541; *p* < 0.001), compared with an OR of 1.484 (95% CI 1.409–1.563) in the complete-case analysis. The association with age was similarly stable (OR per 10-year increase = 1.455, 95% CI 1.335–1.575; *p* < 0.001, versus 1.459, 95% CI 1.336–1.593, in the complete-case analysis). Discrimination was also comparable, with a mean AUC of 0.7552 across the 50 imputed datasets versus an AUC of 0.7589 in the complete-case analysis (Table S5). These findings indicate that the main results were robust to the handling of missing CRP/albumin ratio data.

#### 3.4.4. Internal Validation

Bootstrap internal validation using 1000 nonparametric resamples showed minimal optimism for both mortality models. For 7-day in-hospital mortality, the apparent AUC was 0.7680, with a mean bootstrap optimism of 0.0012 and an optimism-corrected AUC of 0.7668. The optimism-corrected calibration slope and intercept were 0.9950 and −0.0078, respectively. For 30-day in-hospital mortality, the apparent AUC was 0.7589, with a mean bootstrap optimism of 0.0009 and an optimism-corrected AUC of 0.7580. The corresponding optimism-corrected calibration slope and intercept were 0.9962 and −0.0038, respectively. All 1000 bootstrap resamples produced valid estimates. These findings indicated minimal apparent overfitting and good internal stability of both models (Table S3).

## 4. Discussion

A total of 561 patients (15%) died during their hospital stay. Consistent with data from the Italian National Cause of Death Register, managed by the Italian National Institute of Statistics (ISTAT) [77,78], the most frequent cause of death was severe sepsis (49.9%), followed by respiratory failure (11.9%) and respiratory infections with complications (6.4%). The main causes of death were reported for descriptive purposes only; therefore, no inferential statistical comparison was performed. A full breakdown is provided in Table 9.

The present study shows that the CRP/Alb ratio remained significantly associated with both 30-day and 7-day in-hospital mortality after adjustment for chronological age at admission. Adding age to the CRP/Alb-based models resulted in a modest but statistically significant improvement in discrimination for both mortality outcomes, while no significant interaction between chronological age and ln(CRP/Alb ratio) was observed. Furthermore, although the point estimates of the CRP/Alb ratio AUC were numerically lower in older age groups, differences among age-stratified ROC curves were not statistically significant. Taken together, these findings indicate that chronological age provides additional prognostic information but does not appear to significantly modify the association between the CRP/Alb ratio and short-term in-hospital mortality.

Previous studies have consistently demonstrated an association between elevated CRP/Alb ratio and mortality in sepsis, critically ill patients, heart failure, and geriatric emergency populations. However, these studies primarily evaluated whether CRP/Alb predicted mortality, whereas relatively little attention has been paid to whether its prognostic performance is modified by chronological age. The present study therefore extends previous evidence by formally examining age-adjusted discrimination, age-stratified ROC performance, and CRP/Alb-by-age interactions for both 30-day and very early mortality.

The magnitude of the discriminative performance observed in the present study is generally consistent with, and in some settings higher than, that reported in previous investigations, although direct comparisons should be interpreted cautiously because of differences in study populations, clinical settings, outcome definitions, and follow-up periods.

In patients with severe sepsis or septic shock, Kim et al. [55] reported an AUC of 0.621 for the CRP/Alb ratio in predicting 180-day mortality, higher than that of CRP alone (AUC 0.562), whereas Ranzani et al. [56] showed that the CRP/Alb ratio independently predicted 90-day mortality in septic patients. In a medical intensive care unit population, Park et al. [64] reported a more modest AUC of 0.594 for 28-day mortality, although the CRP/Alb ratio remained an independent predictor and performed better than CRP alone. Conversely, in geriatric emergency department patients, Ayrancı et al. [61] found an AUC of 0.723 for the CRP/Alb ratio, compared with 0.642 for the neutrophil-to-lymphocyte ratio (NLR).

More recently, Uluç et al. [70] reported that the CRP/Alb ratio was independently associated with mortality in geriatric respiratory intensive care patients and showed greater sensitivity and specificity than the HALP score, whereas the platelet-to-lymphocyte ratio did not yield a significant mortality cut-off. Against this heterogeneous background, the AUC of 0.733 observed in our study for the CRP/Alb ratio alone in predicting 30-day mortality is within the range reported for hospitalized and geriatric populations. For 7-day mortality, the corresponding AUC was 0.752, indicating moderate discrimination and only a modest numerical increase compared with the 30-day horizon.

Differences across studies may reflect case mix, severity of illness, timing of biomarker assessment, mortality horizon, and differences in CRP/albumin thresholds. Importantly, the present study was not designed to perform a direct head-to-head comparison with NLR, HALP, APACHE II, SOFA, or other prognostic scores; therefore, no conclusion regarding superiority over established biomarkers or clinical prediction tools can be drawn. Rather, our findings extend previous evidence by showing that the CRP/Alb ratio retains a significant association with mortality after adjustment for chronological age, while no statistically significant differences in its discriminative performance were detected across predefined age strata.

Although age is itself a strong predictor of mortality among hospitalized adults, its addition to the CRP/Alb-based model produced a modest but statistically significant increase in discrimination for both outcomes. For 30-day mortality, the AUC increased from 0.733 for the CRP/Alb ratio alone to 0.759 for the model including ln(CRP/Alb ratio) and age (*p* < 0.001). For 7-day mortality, the AUC increased from 0.752 to 0.768 (*p* = 0.005). Thus, chronological age provided additional discriminative information for both mortality horizons, without evidence that it significantly modified the association between the CRP/Alb ratio and mortality.

These findings indicate that the association between the CRP/Alb ratio and mortality remained statistically significant after adjustment for chronological age and may reflect aspects of the acute inflammatory and nutritional state that are relevant to short-term outcome.

Calibration was satisfactory for both mortality horizons. The Hosmer–Lemeshow tests showed no statistically significant evidence of lack of fit for either the 7-day model (χ^2^(8) = 8.74, *p* = 0.365) or the 30-day model (χ^2^(8) = 10.91, *p* = 0.207), with Brier scores of 0.0558 and 0.1095, respectively. Calibration plots based on deciles of predicted risk showed generally close agreement between observed and predicted mortality. In addition, optimism-corrected calibration slopes and intercepts were close to their ideal values for both models, supporting limited apparent overfitting and good internal stability.

A plausible biological interpretation may relate to the dual nature of this biomarker. C-reactive protein reflects the intensity of the acute inflammatory response, whereas serum albumin represents not only nutritional status but also hepatic protein synthesis, metabolic reserve, and the severity of systemic inflammation. Consequently, the CRP/Alb ratio integrates two complementary biological pathways that are both directly involved in the pathophysiology of acute clinical deterioration. Unlike chronological age, which represents a non-specific marker of accumulated mortality risk, the CRP/Alb ratio may more directly reflect the current biological burden imposed by inflammation, catabolism, and metabolic stress.

These observations are consistent with the current concepts of inflammaging, immunosenescence, and metabolic resilience. Older adults are characterized by chronic low-grade inflammation, impaired immune regulation, anabolic resistance, and reduced physiological reserve. Nevertheless, our findings indicate that the prognostic significance of the CRP/Alb ratio remains broadly preserved across the age spectrum. This suggests that the biomarker may reflect the severity of the ongoing systemic insult rather than chronological age alone. In this context, the CRP/Alb ratio may be interpreted as a marker associated with the inflammatory and nutritional response to acute illness, although the present study was not designed to measure biological resilience or biological age directly.

Although the point estimates of the AUC were numerically lower in progressively older age groups, the differences among age-stratified ROC curves did not reach statistical significance. Moreover, no significant interaction between chronological age and ln(CRP/Alb ratio) was observed for either 30-day or 7-day mortality. Accordingly, the present findings do not provide evidence that chronological age significantly modifies the association between the CRP/Alb ratio and mortality. The numerically lower AUC observed among patients aged ≥85 years should therefore be regarded as a descriptive finding rather than evidence of reduced prognostic performance in this age group.

The evaluation of 7-day mortality provides additional information on the prognostic performance of the CRP/Alb ratio across different short-term mortality horizons. For 7-day in-hospital mortality, the CRP/Alb ratio alone showed moderate discrimination (AUC = 0.752), which increased modestly after the addition of chronological age (AUC = 0.768, *p* = 0.005). These findings indicate that admission CRP/Alb retains prognostic information even for events occurring early during hospitalization, although its discriminative performance was broadly comparable to that observed for 30-day mortality.

The similar discrimination observed across the two mortality horizons suggests that the prognostic information conveyed by the CRP/Alb ratio is not restricted to very early events. Because CRP and albumin were measured only at admission, the present findings specifically reflect the prognostic information available at the beginning of hospitalization and do not address whether longitudinal changes in either biomarker might improve prediction over time.

The CRP/Alb ratio nevertheless represents a simple and inexpensive biomarker that may contribute to early risk stratification using routinely available laboratory measurements at hospital admission.

The present study has several strengths. It includes a large real-world cohort of nearly 3800 consecutive hospitalized adults, standardized laboratory measurements obtained at admission, and comprehensive statistical analyses combining ROC curves, age-adjusted prediction models, age-stratified analyses, interaction analyses, calibration assessment, multiple-imputation sensitivity analysis, and bootstrap internal validation. Furthermore, the simultaneous evaluation of 30-day and 7-day in-hospital mortality allowed assessment of prognostic performance across two distinct short-term mortality horizons.

Nevertheless, some limitations should be acknowledged. First, the retrospective single-center design may limit the generalizability of the findings. Second, CRP and albumin were measured only at admission, preventing assessment of their dynamic changes during hospitalization. Third, although the analyses accounted for chronological age, residual confounding related to frailty, functional status, nutritional status, and comorbidity burden cannot be excluded. The present findings should therefore be interpreted specifically in relation to chronological age and should not be taken as evidence that the prognostic association of the CRP/Alb ratio is independent of the broader range of age-associated clinical characteristics.

In addition, missing CRP/albumin data were not randomly distributed with respect to observed clinical outcomes, as patients with unavailable measurements had substantially shorter hospital stays and higher mortality. Although multiple imputation yielded association estimates and discrimination closely comparable to those obtained in the complete-case analysis, the possibility of missing-not-at-random mechanisms related to unmeasured acute clinical circumstances cannot be excluded. Therefore, the multiple-imputation analysis should be regarded as a sensitivity analysis rather than as a definitive correction for potential selection bias.

Finally, although bootstrap internal validation showed minimal optimism and stable calibration slopes, these findings assess internal model stability rather than transportability. The single-center retrospective design remains an important limitation, and external validation in independent and preferably multicenter cohorts is required before the prognostic models can be considered generalizable.

## 5. Conclusions

In conclusion, the CRP/Alb ratio remained significantly associated with both 30-day and 7-day in-hospital mortality after adjustment for chronological age. Its discriminative performance was broadly preserved across age groups, with no statistically significant differences among age-stratified ROC curves and no evidence of significant effect modification by chronological age. The modest but statistically significant improvement in discrimination obtained by adding chronological age suggests that the CRP/Alb ratio and age provide complementary prognostic information. Overall, the CRP/Alb ratio showed moderate discrimination across both short-term mortality horizons and may represent a readily available marker to support risk stratification in hospitalized adults. However, given the retrospective, observational, single-center design of the study and the absence of external validation, these findings do not support its use as a stand-alone clinical decision tool. Prospective multicenter studies incorporating measures of frailty, comorbidity, functional status, and nutritional status are needed to confirm these findings, externally validate the prognostic models, and determine whether the CRP/Alb ratio provides incremental prognostic information beyond established biomarkers and clinical risk scores.

## Figures and Tables

**Table 1:** Clinical features of patients, stratified by sex. Continuous variables are reported as median [interquartile range, IQR]. Effect size r for continuous variables was calculated from the Mann–Whitney U test as |z|/√N.

	Male	Female	Effect Size Cohen’s h	Sig. (99% CI)
**Subjects N (%)**	1913 (50.5%)	1878 (49.5%)	0.02	0.583 (0.562–0.604)
	**Male**	**Female**	**Effect size—r**	**Sig.** **(99% CI)**
**Age at hospitalization,** **median [IQR]**	76 [65–84]	81 [72–87]	0.180	<0.001 (0.000–0.001)
**LOS,** **median [IQR]**	9 [6–14]	9 [6–14]	0.008	0.622 (0.601–0.642)
**Albuminemia,** **median [IQR]**	3.09 [2.58–3.52]	3.06 [2.58–3.47]	0.030	0.079 (0.068–0.091)
**CRP,** **median [IQR]**	43.1 [10.4–126.5]	36.6 [8.7–118.9]	0.030	0.065 (0.055–0.075)
**CRP/Alb ratio,** **median [IQR]**	14.50 [3.04–45.72]	12.37 [2.53–42.88]	0.025	0.130 (0.116–0.144)

**Table 2:** Features of patients stratified by age group. Values are expressed as number (%).

	Male	Female	Effect Size Cohen’s h	Sig. (99% CI)
**Young N (%)** **(<65 years old)**	472 (62.4%)	285 (37.6%)	0.50	<0.001 (0.000–0.006)
**Young-old N (%)** **(65–74 years old)**	407 (57.8%)	297 (42.2%)	0.31	<0.001 (0.000–0.007)
**Old N (%)** **(75–84 years old)**	607 (49.3%)	625 (50.7%)	0.03	0.626 (0.590–0.661)
**Very Old N (%)** **(≥85 years old)**	427 (38.9%)	671 (61.1%)	0.45	<0.001 (0.000–0.004)

**Table 3:** Clinical characteristics of patients stratified by in-hospital survival status. Continuous variables are reported as median [interquartile range, IQR]. Effect size r for continuous variables was calculated from the Mann–Whitney U test as |z|/√N.

	Deceased	Non-Deceased	Effect Size Cohen’s h	Sig. (99% CI)
**Subjects N (%)**	561 (15%)	3230 (85%)	1.56	<0.001 (0.000–0.001)
	**Deceased**	**Non-Deceased**	**Effect size** **Phi Coefficient**	**Sig.**
**Male N (%)**	290 (51.7%)	1623 (49.8%)	0.01	0.552
**Female N (%)**	271 (48.3%)	1607 (50.2%)		
	**Deceased**	**Non-Deceased**	**Effect size—r**	**Sig.** **(99% CI)**
**Age at hospitalization** **median [IQR]**	84 [76–88]	77 [67–85]	0.178	<0.001 (0.000–0.001)
**LOS** **median [IQR]**	9 [4–16]	9 [6–14]	0.023	0.163 (0.148–0.178)
**Albuminemia** **median [IQR]**	2.50 [2.13–2.93]	3.15 [2.71–3.56]	0.310	<0.001 (0.000–0.001)
**CRP** **median [IQR]**	106.5 [43.2–180.6]	31.9 [7.2–105.4]	0.257	<0.001 (0.000–0.001)
**CRP/Alb ratio** **median [IQR]**	42.74 [15.24–81.53]	10.18 [2.11–36.54]	0.284	<0.001 (0.000–0.001)

**Table 4:** Discriminative performance of the CRP/Alb ratio alone and of the age-adjusted model for 30-day and 7-day in-hospital mortality.

Outcome	Model	AUC	95% CI	AUC Comparison *p*-Value
30-day mortality	CRP/Alb ratio alone	0.733	0.712–0.754	
	ln(CRP/Alb ratio) + age	0.759	0.739–0.779	<0.001
7-day mortality	CRP/Alb ratio alone	0.752	0.724–0.781	
	ln(CRP/Alb ratio) + age	0.768	0.740–0.796	0.005

**Note:** The age-adjusted model was based on predicted probabilities derived from multivariable logistic regression including ln(CRP/Alb ratio) and chronological age. AUC, area under the receiver operating characteristic curve; CI, confidence interval; ROC, receiver operating characteristic.

**Table 5:** AUC values from ROC analyses of 30-day mortality and CRP/Alb ratio stratified by age.

Age Categories (Years)	Obs.	AUC	Std. Error	95% CI
**<65**	757	0.763	0.030	0.704–0.823
**65–74**	704	0.749	0.028	0.694–0.804
**75–84**	1232	0.732	0.019	0.695–0.769
**≥85**	1098	0.699	0.018	0.663–0.734

**Table 6:** Age-adjusted logistic regression analysis for 30-day in-hospital mortality and assessment of effect modification by chronological age.

Analysis	Effect Estimate	Sig.	95% CI
**CRP/Alb ratio, per doubling**	OR: 1.484	<0.001	1.409–1.563
**Age, per 10-year increase**	OR: 1.459	<0.001	1.336–1.593
**ln(CRP/Alb ratio) × age interaction**	β −0.0342	0.322	−0.1018–0.0335

**Note:** Analyses were performed in 3791 patients. Main-effects estimates were obtained from the age-adjusted logistic regression model including ln(CRP/Alb ratio) and chronological age. The interaction coefficient was obtained from a separate model additionally including the ln(CRP/Alb ratio) × age interaction term. The effect of the CRP/Alb ratio is expressed per doubling of the ratio, whereas the effect of age is expressed per 10-year increase. β, regression coefficient; CI, confidence interval; OR, odds ratio.

**Table 7:** Age-stratified discriminative performance of the CRP/Alb ratio for 7-day in-hospital mortality.

Age Categories (Years)	Obs.	AUC	Std. Error	95% CI
**<65**	757	0.776	0.044	0.690–0.862
**65–74**	704	0.765	0.040	0.686–0.843
**75–84**	1232	0.766	0.025	0.717–0.816
**≥85**	1098	0.710	0.024	0.663–0.758

**Note:** The overall comparison of age-stratified ROC curves was not statistically significant (χ^2^(3) = 3.52, *p* = 0.318). AUC, area under the receiver operating characteristic curve; CI, confidence interval; ROC, receiver operating characteristic.

**Table 8:** Age-adjusted logistic regression analysis for 7-day in-hospital mortality and assessment of effect modification by chronological age.

Analysis	Effect Estimate	Sig.	95% CI
**CRP/Alb ratio, per doubling**	OR: 1.578	<0.001	1.457–1.710
**Age, per 10-year increase**	OR: 1.331	<0.001	1.180–1.502
**ln(CRP/Alb ratio) × age interaction**	β −0.0357	0.491	−0.1373–0.0659

**Note:** Analyses were performed in 3791 patients. Main-effects estimates were obtained from the age-adjusted logistic regression model including ln(CRP/Alb ratio) and chronological age. The interaction coefficient was obtained from a separate model additionally including the ln(CRP/Alb ratio) × age interaction term. The effect of the CRP/Alb ratio is expressed per doubling of the ratio, whereas the effect of age is expressed per 10-year increase. β, regression coefficient; CI, confidence interval; OR, odds ratio.

**Table 9:** Main causes of death, expressed as Number (%), among study subjects.

	Deceased
Severe Sepsis N (%)	280 (49.9%)
Pulmonary edema and respiratory failure N (%)	67 (11.9%)
Any respiratory infection and inflammation with complications N (%)	36 (6.4%)
Pleural effusion with complications N (%)	25 (4.5%)
Heart failure and shock N (%)	19 (3.4%)
Malignant neoplasms of digestive system with complications N (%)	11 (2.0%)
Severe renal failure N (%)	10 (1.8%)
Any myeloproliferative disorders and poorly differentiated neoplasms with complications N (%)	10 (1.8%)
All other causes N (%)	103 (18.4%)
**Total number of deaths N (%)**	561 (100.0%)

## Data Availability

The raw data supporting the conclusions of this article will be made available by the authors on request.

## Supplementary Materials

The following supporting information can be downloaded at: https://www.mdpi.com/article/10.3390/nu18182988/s1, TRIPOD Checklist: Prediction Model Development; Figure S1: Interaction between metabolic stress, tissue catabolism, and nutritional status. Increased C-reactive protein (CRP) and concomitant hypoalbuminemia may jointly reflect inflammatory burden, tissue catabolism, and impaired nutritional status associated with adverse clinical outcomes (author’s elaboration based on the scientific literature); Figure S2: Calibration plot of the age-adjusted logistic regression model for predicting 7-day in-hospital mortality. Points represent observed mortality proportions plotted against mean predicted probabilities across deciles of predicted risk. The diagonal line represents perfect calibration; Figure S3: Calibration plot of the age-adjusted logistic regression model for predicting 30-day in-hospital mortality. Points represent observed mortality proportions plotted against mean predicted probabilities across deciles of predicted risk. The diagonal line represents perfect calibration; Table S1: Observed and expected 7-day in-hospital mortality across deciles of predicted risk and model calibration statistics; Table S2: Observed and expected 30-day in-hospital mortality across deciles of predicted risk and model calibration statistics; Table S3: Bootstrap internal validation of the age-adjusted logistic regression models for 7-day and 30-day in-hospital mortality; Table S4: Comparison of patients with available and missing CRP/Alb ratio data; Table S5: Sensitivity analysis using multiple imputation for missing CRP/albumin ratio data.
