# Supplementary material for: The C-Reactive Protein/Albumin Ratio Predicts In-Hospital Mortality Across Age Groups
Source: Nutrients. 2026 Sep 12;18(18):2988. doi: 10.3390/nu18182988 (PMC13610636; doi:10.3390/nu18182988)
Supplement: Supplementary file 1 [file nutrients-18-02988-s001.zip › nutrients-4475171-supplementary.pdf]

nutrients

MDPI

Supplementary Materials

TRIPOD Checklist: Prediction Model Development

Section/Topic	Item	Checklist Item	Paragraph
**Title and abstract**			
Title	1	Identify the study as developing and/or validating a multivariable prediction model, the target population, and the outcome to be predicted.	Title
Abstract	2	Provide a summary of objectives, study design, setting, participants, sample size, predictors, outcome, statistical analysis, results, and conclusions.	Abstract
**Introduction**			
Background and objectives	3a	Explain the medical context (including whether diagnostic or prognostic) and rationale for developing or validating the multivariable prediction model, including references to existing models.	Introduction, Background
	3b	Specify the objectives, including whether the study describes the development or validation of the model or both.	Introduction, last paragraph
**Methods**			
Source of data	4a	Describe the study design or source of data (e.g., randomized trial, cohort, or registry data), separately for the development and validation data sets, if applicable.	Methods, Section 2.1 (Study Population)
	4b	Specify the key study dates, including start of accrual; end of accrual; and, if applicable, end of follow-up.	Methods, Section 2.1 (Study Population)
Participants	5a	Specify key elements of the study setting (e.g., primary care, secondary care, general population) including number and location of centres.	Methods, Section 2.1 (Study Population)
	5b	Describe eligibility criteria for participants.	Methods, Section 2.1 (Study Population)
	5c	Give details of treatments received, if relevant.	Not relevant
Outcome	6a	Clearly define the outcome that is predicted by the prediction model, including how and when assessed.	Methods, Section 2.1 and 2.2
	6b	Report any actions to blind assessment of the outcome to be predicted.	Not applicable (retrospective study)
Predictors	7a	Clearly define all predictors used in developing or validating the multivariable prediction model, including how and when they were measured.	Methods, Section 2.1 and 2.2
	7b	Report any actions to blind assessment of predictors for the outcome and other predictors.	Not applicable (retrospective study)

 https://doi.org/10.3390/nu18182988

Sample size	8	Explain how the study size was arrived at.	Methods, Section 2.1 (Study Population)
Missing data	9	Describe how missing data were handled (e.g., complete-case analysis, single imputation, multiple imputation) with details of any imputation method.	Methods, Section 2.3 (Missing Data)
Statistical analysis methods	10a	Describe how predictors were handled in the analyses.	Methods, Section 2.2 (Statistical Analysis)
	10b	Specify type of model, all model-building procedures (including any predictor selection), and method for internal validation.	Methods, Section 2.2 (Statistical Analysis) and 2.4 (Internal validation)
	10c	Specify all measures used to assess model performance and, if relevant, to compare multiple models.	Methods, Section 2.2 (Statistical Analysis) and 2.4 (Internal validation)
Risk groups	11	Provide details on how risk groups were created, if done.	Not done
**Results**			
Participants	13a	Describe the flow of participants through the study, including the number of participants with and without the outcome and, if applicable, a summary of the follow-up time. A diagram may be helpful.	Results, Sections 3.1–3.3; Figure 1
	13b	Describe the characteristics of the participants (basic demographics, clinical features, available predictors), including the number of participants with missing data for predictors and outcome.	Results, Sections 3.1–3.3; Tables 1–3; Section 3.5.3 and Table S4 for missing-data characteristics
Model development	14a	Specify the number of participants and outcome events in each analysis.	Results, Sections 3.3–3.5
	14b	If done, report the unadjusted association between each candidate predictor and outcome.	Unadjusted associations
Model specification	15a	Not reported as an individual risk-prediction equation; the study primarily evaluated prognostic association and discrimination rather than proposing a stand-alone clinical prediction tool.	Results,<br>3.4 Predictive performance
	15b	Not applicable; the models were not intended for direct calculation of individual clinical risk.	Results,<br>3.4 Predictive performance
Model performance	16	Report performance measures (with CIs) for the prediction model.	Results, Sections 3.4–3.5; Tables 4–8; Supplementary Tables S1–S3
**Discussion**			
Limitations	18	Discuss any limitations of the study (such as nonrepresentative sample, few events per predictor, missing data).	Discussion, last paragraphs
Interpretation	19b	Give an overall interpretation of the results, considering objectives, limitations, and results from similar studies, and other relevant evidence.	Discussion, entire section

https://doi.org/10.3390/nu18182988

Implications	20	Discuss the potential clinical use of the model and implications for future research.	Discussion, last paragraphs
**Other information**			
Supplementary information	21	Provide information about the availability of supplementary resources, such as study protocol, Web calculator, and data sets.	Supplementary Materials section
Funding	22	Give the source of funding and the role of the funders for the present study.	Funding section

https://doi.org/10.3390/nu18182988

Figure S1. Interaction between metabolic stress, tissue catabolism, and nutritional status. Increased C-reactive protein (CRP) and concomitant hypoalbuminemia may jointly reflect inflammatory burden, tissue catabolism, and impaired nutritional status associated with adverse clinical outcomes (author's elaboration based on the scientific literature).

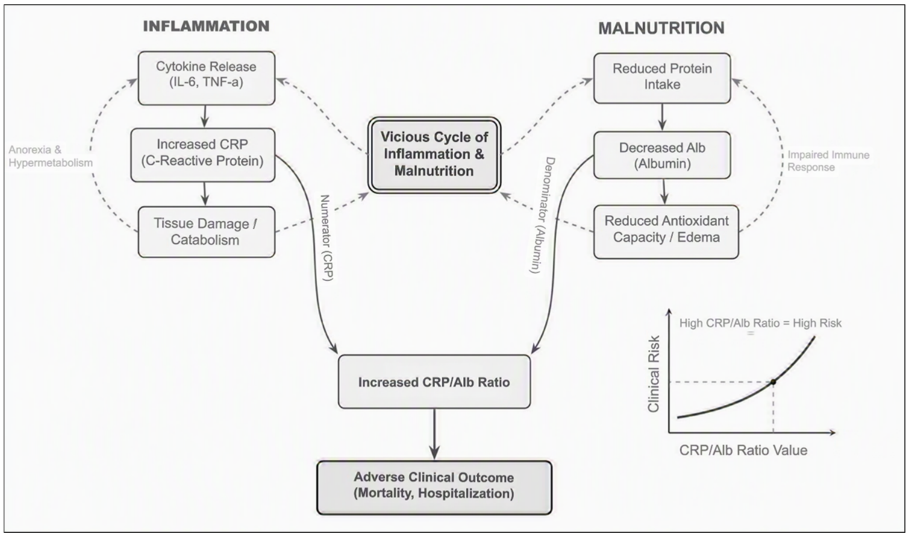

Figure S2. Calibration plot of the age-adjusted logistic regression model for predicting 7-day in-hospital mortality. Points represent observed mortality proportions plotted against mean predicted probabilities across deciles of predicted risk. The diagonal line represents perfect calibration.

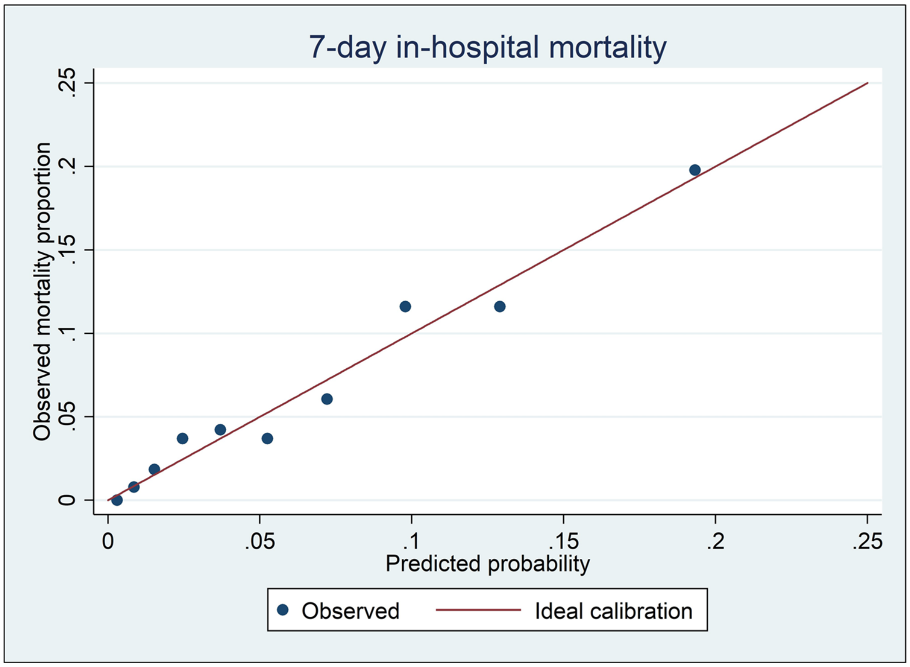

https://doi.org/10.3390/nu18182988

Figure S3. Calibration plot of the age-adjusted logistic regression model for predicting 30-day in-hospital mortality. Points represent observed mortality proportions plotted against mean predicted probabilities across deciles of predicted risk. The diagonal line represents perfect calibration.

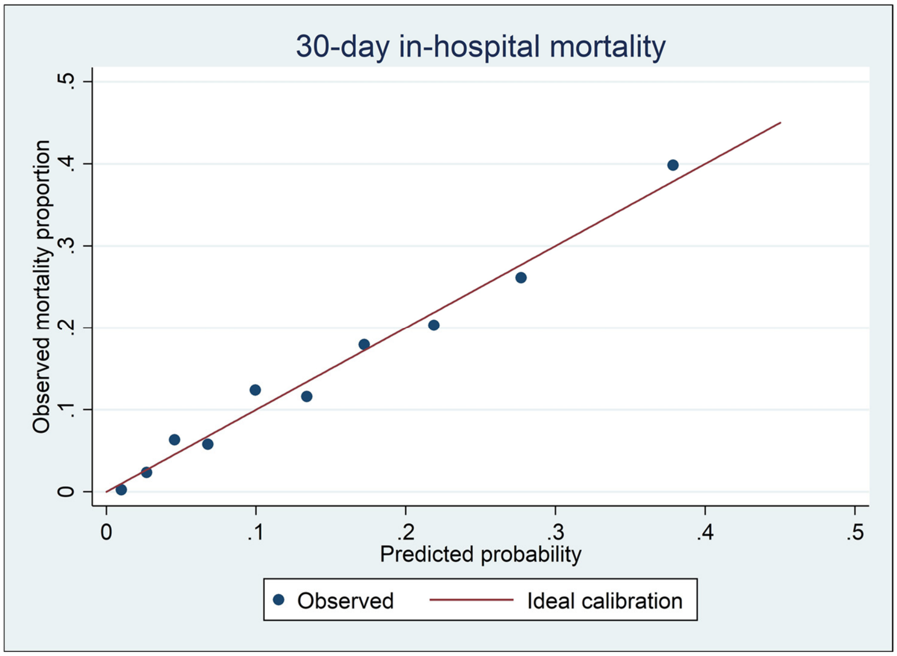

Table S1. Observed and expected 7-day in-hospital mortality across deciles of predicted risk and model calibration statistics.

Groups	Total Observed	Observed deaths (O)	Expected deaths (E)	O – E
**1**	381	0	1.1	−1.1
**2**	378	3	3.2	−0.2
**3**	379	7	5.8	1.2
**4**	379	14	9.3	4.7
**5**	379	16	14.0	2.0
**6**	379	14	19.9	−5.9
**7**	379	23	27.3	−4.3
**8**	379	44	37.1	6.9
**9**	379	44	48.9	−4.9
**10**	379	75	73.3	1.7

Number of observations = 3,791;
Hosmer–Lemeshow $\chi^2(8)$ = 8.74, p = 0.365;
Brier score = 0.0558.

Note: Expected deaths were derived from the age-adjusted logistic regression model including ln(CRP/Alb ratio) and chronological age. O − E indicates the difference between observed and expected deaths. The Hosmer–Lemeshow goodness-of-fit test was calculated across deciles of predicted risk.

https://doi.org/10.3390/nu18182988

Table S2. Observed and expected 30-day in-hospital mortality across deciles of predicted risk and model calibration statistics.

Groups	Total Observed	Observed deaths (O)	Expected deaths (E)	O – E
**1**	380	1	3.8	−2.8
**2**	379	9	10.2	−1.2
**3**	379	24	17.2	+6.8
**4**	379	22	25.7	−3.7
**5**	379	47	37.7	+9.3
**6**	379	44	50.7	−6.7
**7**	379	68	65.3	+2.7
**8**	379	77	82.9	−5.9
**9**	379	99	105.0	−6.0
**10**	379	151	143.4	+7.6

Number of observations = 3,791;
Hosmer–Lemeshow $\chi^2(8)$ = 10.91,
p = 0.207;
Brier score = 0.1095.
Note: Expected deaths were derived from the age-adjusted logistic regression model including ln(CRP/Alb ratio) and chronological age. O − E indicates the difference between observed and expected deaths. The Hosmer–Lemeshow goodness-of-fit test was calculated across deciles of predicted risk.

Table S3. Bootstrap internal validation of the age-adjusted logistic regression models for 7-day and 30-day in-hospital mortality.

Performance metric	7-day mortality model	30-day mortality model
Number of observations	3,791	3,791
Apparent AUC	0.7680	0.7589
Mean bootstrap optimism	0.0012	0.0009
Optimism-corrected AUC	**0.7668**	**0.7580**
Optimism-corrected calibration slope	**0.9950**	**0.9962**
Optimism-corrected calibration intercept	**−0.0078**	**−0.0038**
Bootstrap resamples	1,000	1,000

Note: Internal validation was performed using 1,000 nonparametric bootstrap resamples. In each bootstrap sample, the logistic regression model including ln(CRP/Alb ratio) and chronological age was refitted, and its performance was evaluated in both the bootstrap sample and the original dataset. Optimism was calculated as the difference between bootstrap-sample and original-sample performance, and the apparent AUC was corrected by subtracting the mean bootstrap optimism. Optimism-corrected calibration slope and intercept were derived from the calibration model obtained by applying each bootstrap-fitted model to the original dataset. AUC, area under the receiver operating characteristic curve.

https://doi.org/10.3390/nu18182988

Table S4. Comparison of patients with available and missing CRP/Alb ratio data.

Characteristic / analysis	Complete cases	Missing CRP/Alb data	P value
N	3,791	145	—
Age, years, median [IQR]	78 [68–86]	78 [68–86]	0.779
Female sex, n (%)	1,878 (49.5)	73/144 (50.7)	0.785
LOS, days, median [IQR]	9 [6–14]	2 [1–7]	<0.001
30-day in-hospital mortality, n (%)	542 (14.3)	79 (54.5)	<0.001

Note: Continuous variables are presented as median [interquartile range] and were compared using the Mann–Whitney U test. Categorical variables were compared using Pearson's chi-square test. Sex was unavailable for one patient in the group with missing CRP/albumin ratio data. CRP, C-reactive protein; IQR, interquartile range.

Table S5. Sensitivity analysis using multiple imputation for missing CRP/albumin ratio data.

Performance measure	Complete-case analysis	Multiple-imputation analysis
N	3,791	3,936
CRP/Alb ratio, OR per doubling (95% CI)	1.484 (1.409–1.563)	1.466 (1.391–1.541)
Age, OR per 10-year increase (95% CI)	1.459 (1.336–1.593)	1.455 (1.335–1.575)
AUC	0.7589	0.7552*

Note: Multiple imputation was performed using 50 imputed datasets. Odds ratios from the multiple-imputation analysis were combined using Rubin's rules. *The AUC for the multiple-imputation analysis represents the mean AUC across the 50 imputed datasets and was not pooled using Rubin's rules. CRP, C-reactive protein; OR, odds ratio; CI, confidence interval; AUC, area under the receiver operating characteristic curve.

https://doi.org/10.3390/nu18182988
